# Experimental evidence suggests that specular reflectance and glossy appearance help amplify warning signals

**DOI:** 10.1038/s41598-017-00217-5

**Published:** 2017-03-21

**Authors:** Samuel J. Waldron, John A. Endler, Janne K. Valkonen, Atsushi Honma, Susanne Dobler, Johanna Mappes

**Affiliations:** 1 Centre of Excellence in Biological Interactions, University of Jyväskylä, Department of Biological and Environmental Sciences, PO Box 35, 40014 University of Jyväskylä, Jyväskylä, Finland; 2 Molecular Evolutionary Biology, Department of Biology, Universität Hamburg, Martin-Luther-King Pl. 3, 20146 Hamburg, Germany; 3 Centre for Integrative Ecology, School of Life and Environmental Sciences, Deakin University, Waurn Ponds, Victoria, 3216 Australia; 4 Department of Ecosystem Studies, School of Environmental Science, The University of Shiga Prefecture, 2500, Hassaka-cho, Hikone City, Shiga 522-8533 Japan

## Abstract

Specular reflection appears as a bright spot or highlight on any smooth glossy convex surface and is caused by a near mirror-like reflectance off the surface. Convex shapes always provide the ideal geometry for highlights, areas of very strong reflectance, regardless of the orientation of the surface or position of the receiver. Despite highlights and glossy appearance being common in chemically defended insects, their potential signalling function is unknown. We tested the role of highlights in warning colouration of a chemically defended, alpine leaf beetle, *Oreina cacaliae*. We reduced the beetles’ glossiness, hence their highlights, by applying a clear matt finish varnish on their elytra. We used blue tits as predators to examine whether the manipulation affected their initial latency to attack, avoidance learning and generalization of warning colouration. The birds learned to avoid both dull and glossy beetles but they initially avoided glossy prey more than dull prey. Interestingly, avoidance learning was generalized asymmetrically: birds that initially learned to avoid dull beetles avoided glossy beetles equally strongly, but not *vice versa.* We conclude that specular reflectance and glossiness can amplify the warning signal of *O. cacaliae*, augmenting avoidance learning, even if it is not critical for it.

## Introduction

When light reaches the surface of an object it can be reflected as a beam or scattered out in many directions depending upon the surface properties. For example, fine-scale rough surfaces scatter light in many directions, reducing its directional reflection intensity, whereas smooth (shiny or glossy) surfaces reflect light like a mirror predominately in one direction, causing a high intensity, specular reflection or highlight^1^. Highlights, the bright spots that are normally associated with glossiness and a convex surface shape, are present even when the illuminant angle is extremely acute^2^. If a surface is convex, the viewing position of the receiver relative to the surface has little affect on the presence or intensity of specular reflectance because there is always a part of the convex surface at the correct angle to reflect to the viewer^2^. This is true even for interference colours which have maximum reflectance at a particular set of illuminant and viewing angles. In that case the highlight has the maximum effect of constructive interference and will be fringed with other colours at lower intensities. Many chemically defended beetle species (and other insect species such as some Heteroptera species) have convex glossy body surfaces producing a reflectance highlight. The glossy appearance is not normally associated with cryptic species, although some glossy insects have been suggested to resemble water droplets^3, 4^. These highlights are likely to contrast greatly against background foliage and the rest of the body, increasing their overall conspicuousness or salience.

Aposematic species often advertise their defences with conspicuous warning signals that allow predators learn to associate unprofitability with conspicuousness^5^. It is therefore possible that because of its high luminance, specular reflectance can have or aid a warning signal function. The possible importance of luminance contrast in predator learning and initial avoidance of defensive signalling has been suggested (e.g. refs 6, 7 and 8). For example, Prudic *et al.* ^9^ tested the effect of luminance on invertebrate predators by painting milkweed bugs different tones of grey and found that a contrast in luminance was capable of being an effective warning signal. Maan and Cummings^10^ found a positive relationship between luminance (using avian perceptual models) and toxicity in the strawberry poison frog (*Dendrobates pumilio*). This suggests that luminance could play a significant role in aposematic signalling. However, there has been no attempt to relate the importance of specular reflectance to warning signalling.

**Figure 1:**
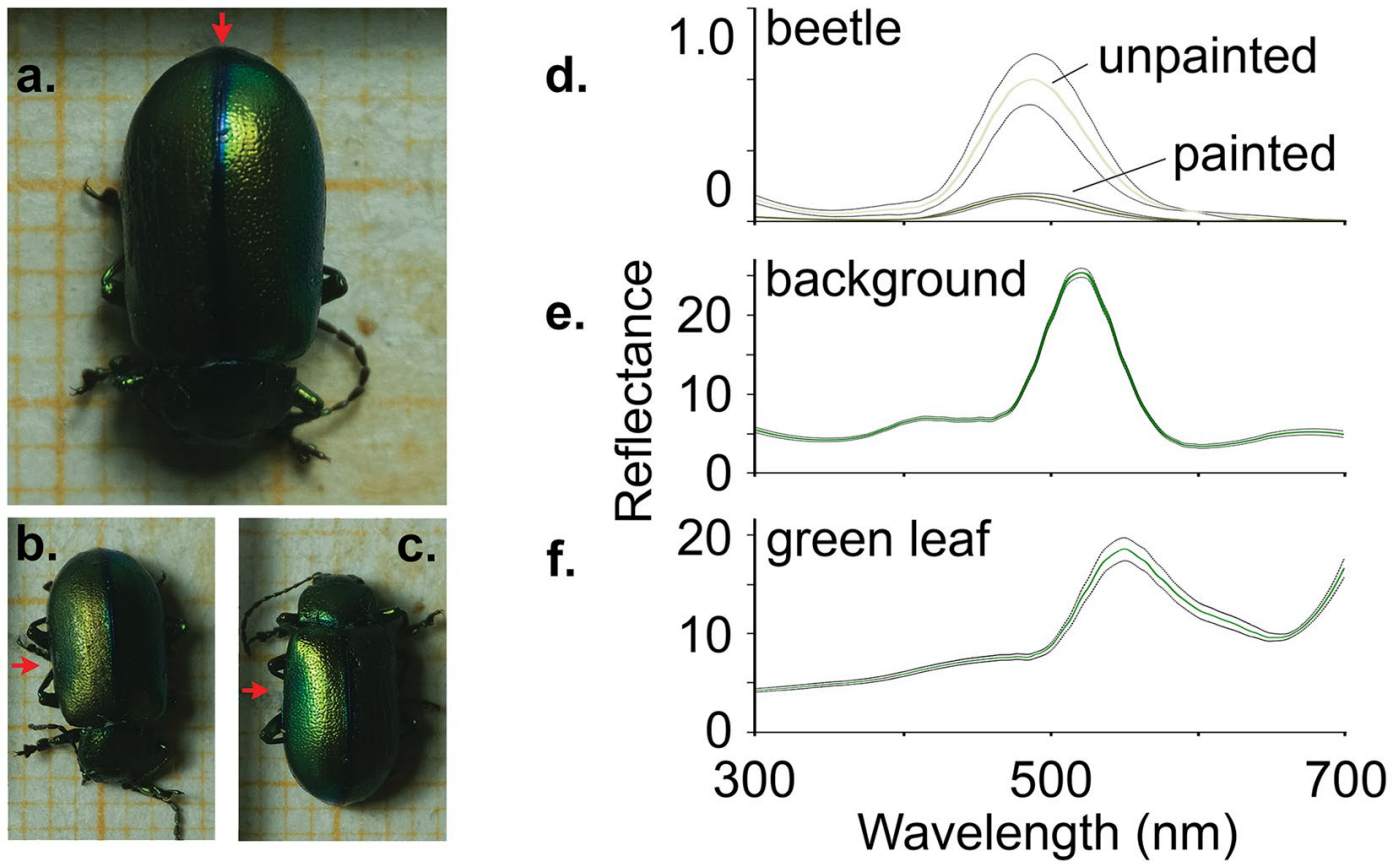
For illustrative purposes the *O. cacaliae* (green phenotype) beetle shown here was manipulated by painting the right-hand side elytron only with a matt clear-coat varnish. One square on the background paper represents 1 mm^2^. In the experiment, the whole beetle was either painted or left untouched (see section 2(c)). A beetle was photographed from above with the incident light (light source) positioned at an elevation of 80° (red arrows indicate the direction of the light) and an azimuth of 180° from the beetles head. The beetle was positioned with its posterior towards the light source (**a**) and then with the right (**b**) and the left (**c**) hand sides facing towards the light. Note the specular reflection does not appear or is greatly reduced after manipulation (left side of beetle in panel **b**). The elytra appear almost matt black when not in favourable lighting geometries suitable to reflect light. (**d**) Standardized (between zero and one) average reflectance of three manipulated (dark green line) and non manipulated (light green line) beetle elytra (black dotted lines represents ± standard deviation) measured in their maximum reflectance angles. (**e**) Reflectance spectrum of the green background placed in the petri dishes used during the behavioural assays (**f**). Reflectance spectrum of a green leaf.

To assess the possible significance of specular reflectance on aposematism we tested the effect of degree of glossiness on predator behaviour on *Oreina cacaliae* beetles. We reduced the glossiness of these beetles and hence their directional luminance (defined as the intensity of light emitted in a given direction from a surface per unit area) and increased the diffuse reflectance of their elytra. *Oreina cacaliae* (Fig. 1) have highly reflective convex elytra. They feed exposed on their host plants (Asteraceae) and utilize sequestered pyrrolizidine alkaloids as defensive compounds^11^. Together with their green structural colouration, conspicuous reflective highlight and bold behaviour^12^ (Fig. 1, Supplementary Fig. S1) it has been suggested that *O. cacaliae* are aposematic^12–14^. For these reasons *O. cacaliae* presents the perfect system to investigate the role of specular reflectance for warning signalling. Using wild-caught blue tits (*Cyanistes caeruleus*) as predators and *O. cacaliae* as prey we measured (a) predators’ willingness to attack, (b) speed of avoidance learning and (c) generalization for attacking prey with natural and reduced specular reflectance and glossiness.

## Material and Methods

### Birds and Beetles

All experimental methods were carried out in accordance with guidelines and regulations of the Animal welfare-body of University of Jyväskylä and the Finnish Act on Animal Experimentation. All experimental protocols were approved by National Experimental Animal Committee (Permit number: ESAVI-2010-087517Ym-23) and Central Finland Centre for Economic Development, Transport and the Environment (Permit number: ESELY/1017/07.01/2010). We adhered to Finnish and German legislation, and followed the accepted ethical guidelines of the scientific community including its journals.

In total 38 blue tits (*Cyanistes caeruleus*) used in the predator behaviour assays were caught between January and March 2012 at feeding sites in Konnevesi Research Station, Central Finland, where the experiments were conducted. The birds were individually housed in purpose-built aviaries during the experimental period (food and water provided *ad libitum*) and when experiments were complete they were released at the place of capture. Most birds were kept in captivity less than a week. We used blue tits as predators because it is a well-established model species in predation experiments^15, 16^, its vision system is known^17^ and it co-occurs with Oreina beetles in the Alps (personal observations SW, SD, JM). Furthermore, as an omnivorous predator it is likely to be sensitive to defensive toxins thus enabling avoidance learning.

Green *Oreina cacaliae* beetles used as prey in the predator behaviour assay were collected near Zastler in the Black Forest, Germany, (July, 2011). The defensive potential of any *O. cacaliae* secretion depends on availability of pyrrolizidine alkaloids in their host plants, frequency of recent attacks and subsequent secretion of compounds before and during capture. Before freezing (−80 °C), the beetles were provoked into releasing any remaining defensive secretions by poking their head with forceps to standardize their individual chemical content, distastefulness and potential olfactory signal. The beetles were then cleaned with 50% ethanol and dried yet still possessed a significant amount of pyrrolizidine alkaloids in their haemolymph^14^.

### Reflectance manipulation and measurements

To reduce the specular reflection and shininess of the beetles’ we painted their elytra with a matt finish clear-coat varnish (Fig. 1) normally used as a protective layer over car paint. The varnish was spectrally flat except at the UV range, but there was almost no UV in the lights used in the experiment (Supplementary Figs S2 and S3). The matt finish increased the scattering of reflected light and markedly decreased directional reflection, hence making beetles less glossy and therefore strongly reducing the highlight intensity. The elytra of three beetles were used to estimate the reflection spectral effects of the manipulation used in the predator behaviour assay. The reflectance spectra (Fig. 1d) represent only the direct reflectance because the directional probe arrangement does not receive diffuse light. The direct light decreases because the matt finish produces a lot more diffuse reflectance than the original glossy surface. In real life, the viewing geometry refers to the position of the surface (beetle elytron) in relation to the angles of the sun and receiver, in this case an approaching predator. Analysis was based on the highly repeatable maximum reflectance (λ_max_) methodology as described in Meadows *et al.* ^18^ (see below).

All spectra were collected using a Maya 2000 Pro spectrometer and D2000 – Deuterium, UV Light Source that were connected to optical fibres (QP200-2-SR-BX, Ocean Optics). To ensure steady and repeatable positioning of beetle elytra during spectrometer measurements, small sections (*ca* 4 mm^2^) were cut out of each elytron and used for measurements. Quartz collimating lenses were connected to the ends of both optical fibres to transfer the light to and from the elytra. Lenses were focussed to create an illuminated spot diameter of *ca* 3.5 mm and measurement spot diameter of *ca* 2 mm. As we were interested in how our manipulation affected the specular reflectance of the beetle elytra we only measured the directional rather than the total reflectance. All spectrometer measurements were made in a dark room, where the collimated light source was the only available light. Spectra were expressed relative to a 99% white reflectance standard (Labsphere). Each segment was measured three times and averaged; variation among the three measurements was minimal with an Intraclass Correlation Coefficient (ICC) of 0.98 (95% Confidence Intervals: upper = 1.01, lower = 0.95)^19^. An angle-dependent reflectance (ARM) apparatus, built at Arizona State University^18^, was used to allow accurate measurements of viewing geometry (see Supplementary Fig. S4).

**Figure 2:**
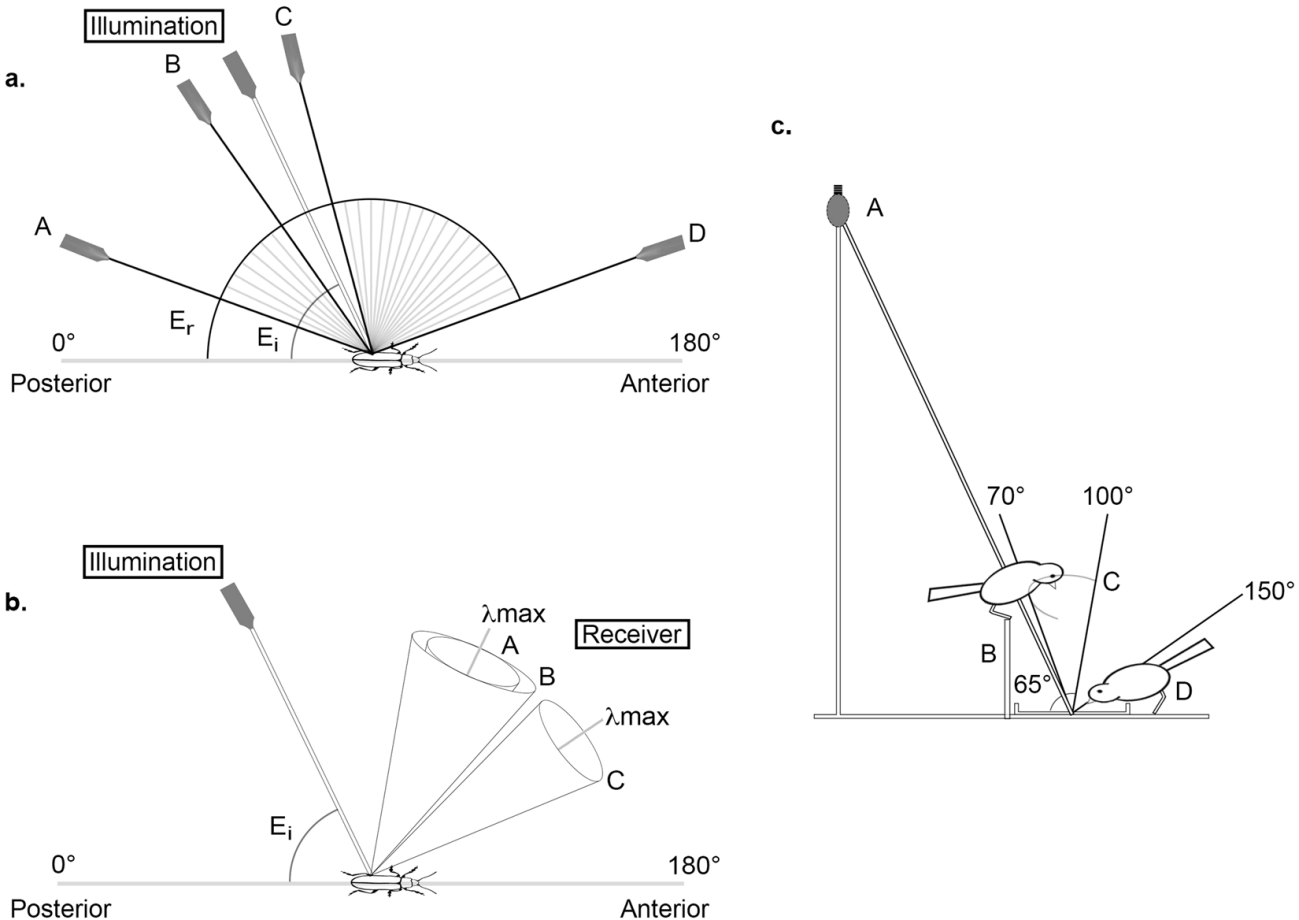
(**a**) We measured the effect of the manipulation at multiple angles. Birds and beetles are active during daylight hours. We therefore fixed the illumination at 65° that corresponds to the maximum elevation of the sun in mid-July at the site and time of collection (E_i_). We then rotated the receiver in 5° increments from an elevation of E_r_ = 20° (A) −160° (D) mimicking variable approaching directions of potential predators. The light grey lines indicate the spread of each of the receivers’ (E_r_) measurements. The receiver arm is not able to measure between geometries (B) and (C) due to the fixed illumination arm, hence there is a gap in the measurements between angles E_r_ = 55°–75°. (**b**) The reflection properties of a structural surface (e.g. *Oreina* elytra) can be described as a diffuse cone of reflected light around the maximum reflection (λ_max_) of the surface^19^. After manipulation the directional properties of the surface could have either (A) stayed the same (B) changed in size, for example become more diffuse or (C) it could shift. (**c**) The predicted viewing geometry experienced by a bird during the experiments. The incident light (A) within the aviary is at an elevation of 65° in relation to the position of the beetle. After a bird landed on the barrier (B) it typically investigated the prey by moving approximately within the light grey arc (C) this creates a potential range of viewing geometries between 70°–100°. The viewing geometries change again if the bird approaches and/or attacks the beetle on or near the petri dish (D).

As a predator’s approach towards its prey is unpredictable and can change throughout the encounter, the azimuth angle of the receiver (E_r_) was rotated in 5° increments, relative to the beetles long axis starting from an elevation of E_r_ = 20°–160°, to mimic possible predator approach angles (Fig. 2). The elevation of the illumination (E_i_) was fixed at 65°, the maximum elevation for the sun at the time of mid-July when beetles were collected. The beetle was orientated at 0° in relation to the azimuth of the illumination (see Supplementary Fig. S5). While we make significant inroads into analysing and describing the angle dependant appearance of the beetles, our aim here is only to show how the matt clear-coat manipulation has altered the beetle’s appearance in a potential, real world scenario.

### Avian visual modelling

We calculated the Just Noticeable Differences (JND) scale between manipulated and non-manipulated elytra in both chromatic and achromatic channels in both standard daylight (D65) and experimental light conditions (for the irradiance spectrum see Supplementary Fig. S2). Our model used the well-established method for estimating contrasts between two colour patches^20^ using spectral reflectance data and the estimated sensitivities of the blue tit's visual system^17^. Using the PAVO package^21^ in R^22^ we first estimated photoreceptor quantum catch sensitivities for the four single cones (chromatic) and double cones (achromatic) before correcting for receptor noise^20^. The spectra from the green cardboard background used in the experiment were used in the Von Kries correction. The same equipment was used (see section 2(b) and Fig. 1e for the spectrum) to take three measurements at the viewing geometry responsible for the maximum reflectance of each individual beetle elytra (E_i_ = 65° and E_r_ = 115° or 120°) (see Supplementary Table S1). Before applying the visual model, spectral reflectance data were binned in 1 nm increments between 300–700 nm. JNDs were then calculated using the photon catch and the receptor noise models. The tetrachromatic visual system phenotype was used with a Weber fraction coefficient of 0.05.

### Predator behaviour assay

The experimental masonite (hardboard) cages, (50 × 65 × 45 cm) were lit to simulate daylight using a 26 W, Repti Glo 5.0 UVB Compact light bulbs mounted in the centre of the aviary ceiling (see Supplementary Fig. S6). While the Repti Glo light bulb emits UV, the amount of UV recorded at the base of the experimental aviary, where the beetles were exposed to the birds during the experiment, was negligible (see Supplementary Fig. S2). Birds participated in experimental trials one at a time. In total, 38 blue tits were trained to sit on the perch and to anticipate food from behind the barrier. A Petri dish with green cardboard covering the bottom (see the reflectance spectrum from Fig. 1e) was used to present the sunflower seeds during training and beetles during experimental trials. Food was only introduced to the experimental aviary when the bird was settled on the perch. This ensured that the bird’s initial view of the beetle was controlled and on the same plane (posterior-anterior) as that used in the reflectance analysis. The bird’s landing on the barrier indicated the start point to begin recording attack latency (see below). After landing on the barrier, the bird would either lean out over the beetle or crouch low against the barrier to investigate the beetle before approaching. We estimated that the birds would have initially seen the beetle at angles between E_r_ = 70°–100° (Fig. 2c). These angles would have varied throughout the experiment (Fig. 2c) (see Supplementary Table S2 for JND comparisons). Training was completed on day 1 of the experiment. Each bird completed four learning trials on consecutive days and one generalization test on day 5. The birds were randomly split into two groups (N = 16 respectively); the control group received glossy, non-manipulated beetles during the learning stage (days 1–4) and were then offered a dull, manipulated beetle on day 5 to test for generalization between treatments. The treatment group received dull, manipulated beetles on days 1–4 and a glossy beetle (non-manipulated) on day 5. Each trial followed the same protocol: the birds were food-deprived for *ca.* 2 hours before each trial and were then offered a mealworm larva (*Tenebrio molitor*) to ensure they were motivated to forage. This was followed by a food deprivation period of 20 minutes after birds were offered a *T. molitor* beetle to ensure that the birds did not learn to avoid beetles in general. After *T. molitor*, they were offered an *O. cacaliae* beetle (glossy or dull) and then a mealworm larva to ensure the birds was still motivated to forage. This was important in particular if birds refused to attack *O*. *cacaliae*. Attack latency was recorded from when the bird landed on the barrier until it attacked the prey. The birds were given a maximum of 10 minutes to attack the prey before the trial was terminated.

To ensure that the clear varnish coat *per se* does not deter the birds, we ran a separate control assay before the experiment. Using birds (N = 6) that had no previous experience of the clear coat manipulation, we offered manipulated and non-manipulated adult *T. molitor* beetles sequentially, alternating the order. The mean attack latency for non-manipulated *T. molitor* beetles was 4.3 seconds and 3.5 seconds for the clear coat manipulated *T. molitor* beetles; there was no significant difference between the two treatments (Mann-Whitney U test, *U* = 10, *p* = 0.59). Additionally, the birds readily ate all manipulated and non-manipulated beetles. Thus, the clear coat *per se* (potential odour or taste) did not affect the attack or consumption of prey.

Differences in the birds initial willingness to attack glossy, un-manipulated and dull, manipulated *O. cacaliae* beetles was examined using a Cox model^23^ where the risk to be attacked within a time unit was modelled as a function of manipulation. Attack latency over all the learning trials (1 to 4) was examined with a mixed effects Cox model where the risk to be attacked within a time unit was modelled as a function of treatment, trial number (1 to 4) and their interaction (to examine if learning is symmetrical between groups). Bird identity was included in the analysis as a random effect to count for repeated measurements for each bird. To test whether birds generalize their learned avoidance, attack latencies between the last learning trial (trial 4) and test trial (trial 5) were compared separately for treatment groups with a mixed effects Cox model. Again bird identity was included as a random effect. All the analyses were conducted with R-studio (version 0.98.1074) and the coxme package^22, 23^.

## Results

### Manipulation of beetle reflectance

**Figure 3:**
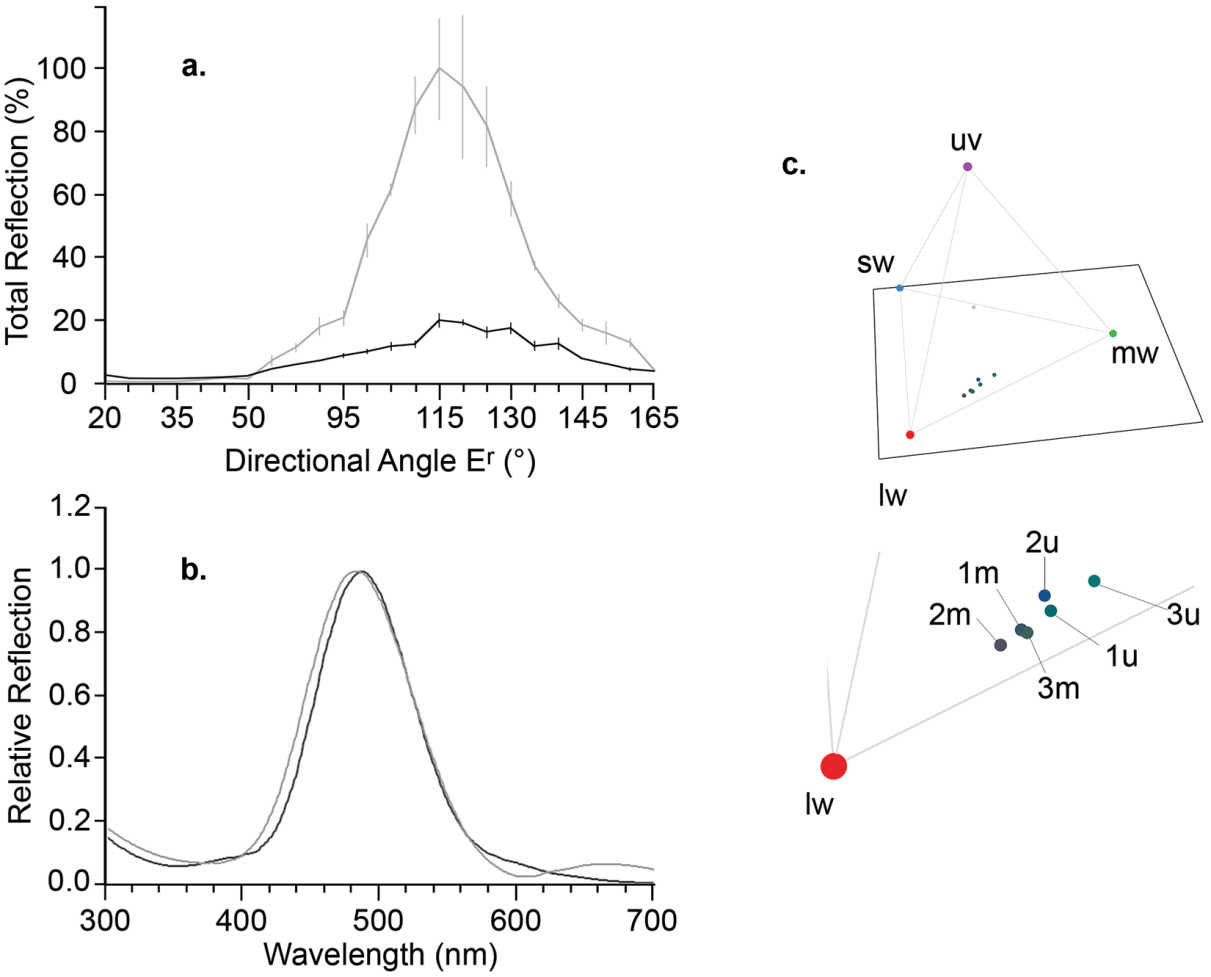
(**a**) Three individual beetles were randomly selected and their mean (±s.e.) values for total reflectance (%) were calculated before (light grey lines) and after (dark grey) manipulation. Note the maximum total reflection for both manipulated and non-manipulated beetles were achieved at E_r_ = 115°. The angle of the illumination (E_i_) was fixed at 65° and the angle of the receiver (E_r_) was rotated from 20° to 160°, angles between 55° and 75° were not measured due to the position of the illumination. The elytra appear matt black between angles 20° and 50° due to unfavourable geometries for reflecting light. (**b**) A comparison of the relative reflectance spectra at E_r_ = 115° (Δλ_max_) before and after the manipulation of the beetle. The spectra were divided by their own totals to make the maximum of each spectrum 1 to allow a direct comparison of the spectral shape. The dark grey line represents the non-manipulated and the light grey line represents the manipulated elytra. (**c**) A representation of each elytron measured before and after manipulation was plotted in a four-dimensional colour space. The axes on the tetrahedron have been weighted according to the cone sensitivity of blue tits in the visual system (see section 2(C)). SW, MW, LW and UV indicates short, medium, long and ultraviolet wavelengths respectively. Labels 1u–3u represent each one of the three individual elytra measured before manipulation (u = unmanipulated), 1 m–3 m corresponds to the respective elytra after manipulation (m = manipulated).

At the maximum reflectance angle of 115°, the manipulation reduced the total directional reflectance by 80% (Fig. 3a). The essential element in the manipulation was its reduction of specular reflection, an increase in the diffuse component of reflection, and its small effect on the relative spectral shape when the surface was measured at its maximum reflection angle (Fig. 3b). This indicates that the absorbance of the varnish (see Supplementary Fig. S3) affected only slightly on the overall “colour” of the beetle’s specular reflectance and predominantly its intensity was affected. The tetrahedral colour plot (Fig. 3c) also supports this assumption as the data points are relatively clustered. The angle of maximum reflection (Δλ_max_) changed within two of the three elytra after manipulation but not consistently in one direction and never more than 5° (see Supplementary Table S1), indicating that the centre of the directionality cone (Fig. 2b) has stayed the same. Furthermore, the total reflection (Fig. 3a) was almost flat between angles 20° and 50° for all beetles and then either rose sharply between 95°–115° (non-manipulated) or steadily between 80°–115° (manipulated) until reaching Δλ_max_. This means the manipulation has suppressed the specular reflection because the directionality cone has become more diffuse (see Fig. 2b, example B).

To quantify the manipulation of beetle colouration, as perceived by the visual system of our avian predator, we compared the same region of the beetle elytra at Δλ_max_ before and after manipulation as comparison patches in the visual model. JNDs for both the chromatic and achromatic channels exceeded the threshold (1 JND) over which they are likely to be discriminable^20^. Differences in JNDs were similar in our experimental light conditions and in standard daylight (D65) (Table 1).

**Table 1:** Patch comparisons between glossy, non-manipulated and dull, manipulated elytra at Δλ_max_.

Elytra	Chromatic	ANGLE (°)	Achromatic	ANGLE (°)
Avian Visual Model using Experimental Conditions				
Elytra 1	7	115	2	115
Elytra 2	14	115	4	120
Elytra 3	14	120	8	115
MEAN	11		5	
SE	2		2	
Avian Visual Model using D65				
Elytra 1	9	115	6	115
Elytra 2	15	115	11	120
Elytra 3	15	120	15	115
MEAN	13		11	
SE	2		3	

Values are given as JNDs and the standard error (SE) of the mean.

### Behavioural Assay

**Figure 4:**
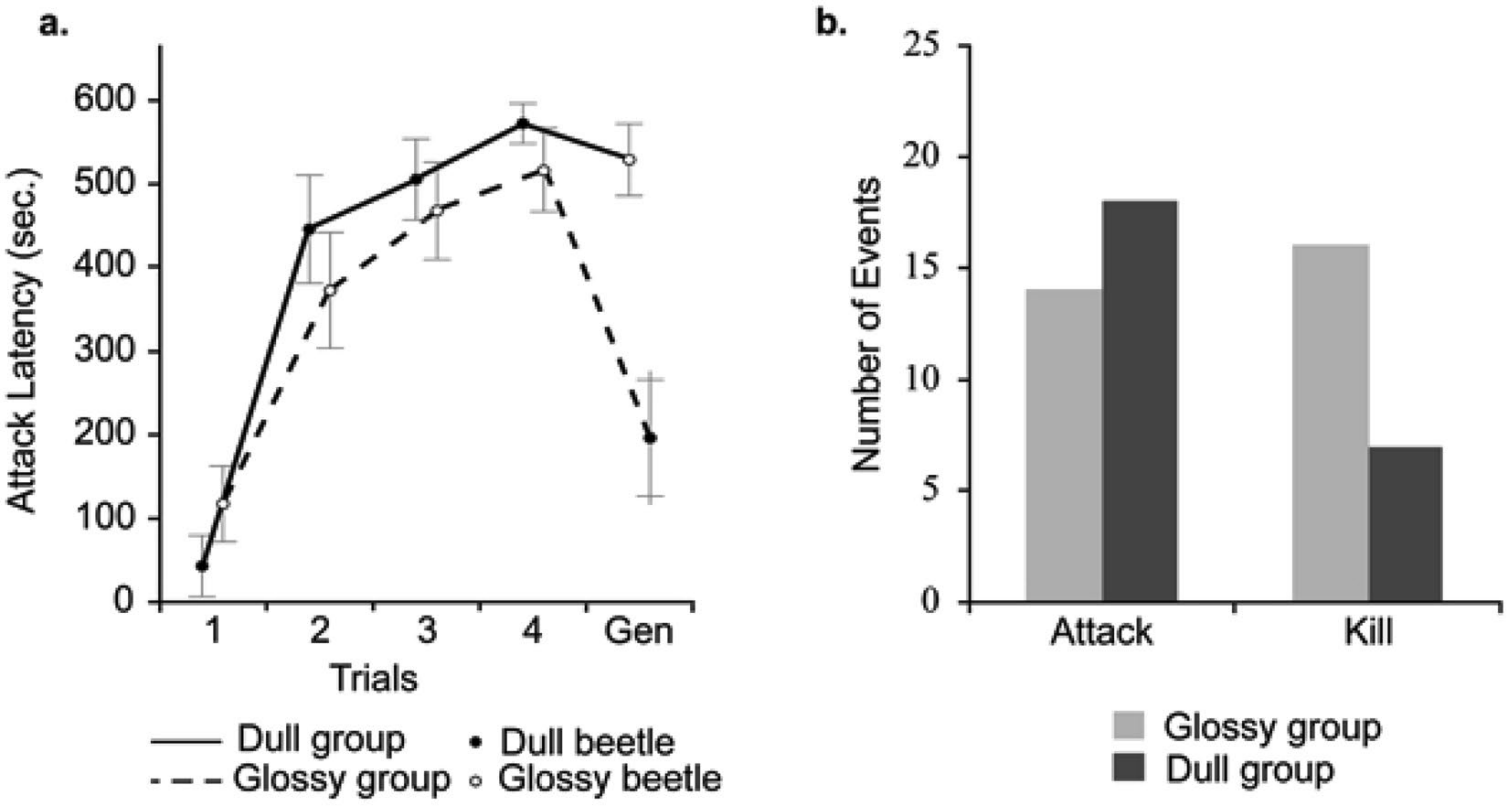
(**a**) Birds’ latency (±s.e.) to attack. Trial 1 measures the reaction during the bird’s first encounter with the beetles. Trials 1–4 measure the bird’s ability to learn the beetles are unpalatable. Generalisation test (Gen) measures transference of the previously learned response (Trials 1–4 with glossy or dull prey) to the alternative prey. Similar attack latency in trials 4 and 5 indicates generalisation. (**b**) The combined number of attacked and killed (attacked with force enough to expose beetles hemolymph) beetles from both treatments in trials 1 to 4.

In the first trial, the birds exhibited a stronger hesitation for glossy beetles compared to dull (varnished) beetles (exp. coef. = 0.415, *z* = −2.3, *p* = 0.021, Fig. 4a). A decrease of the attack probability within time unit between trials 1 to 4 was highly significant for both treatments indicating a strong learning effect (exp. coef. = 0.339, *z* = −4.25, *p* = <0.001 glossy: exp. coef. = 0.253, *z* = −4.25, *p* = <0.001, Fig. 4a) with the biggest difference between trials 1 and 2 (Fig. 4a). After trial 2, the hesitation did continue to increase but not so steeply. Nevertheless, the hesitation delay of over 5 minutes in the last learning trial (Trial 4) for both treatments clearly indicates the birds did not like *O. cacaliae* beetles. Indeed, these beetles are very unpalatable as all birds exhibited either bill wiping or head shaking behaviour, or multiple visits to drink water, after encountering the beetles. However, all *T. molitor* beetles were attacked and consumed without any aversive behaviour. The interaction of treatment and trials for learning rate (Trials 1–4) was significant (exp. coef. = 1.94, *z* = 1.97, *p* = 0.048, Fig. 4a), but the effect was due to the difference between the first and second trials: the interaction disappeared and there was also no main effect of treatment on attack probability (exp. coef. = 1.06, *z* = 0.11, *p* = 0.91) when analysing trials 2–4 only.

All of the birds landed on the petri dish to examine the beetle at least once during the experiment. Regardless of appearance, the birds were very likely to attack the beetles during the first encounter, only 1 bird in each treatment refused to attack the beetle in trial 1. However, during the fourth trial, only 2 out of 16 dull (manipulated) and 4 out of 16 glossy (non-manipulated) beetles were attacked (Table 2). It is worth noting that only one beetle (in the dull group) was consumed during the whole experiment. When the birds decided to attack (after a long hesitation), they appeared to attack glossy unmanipulated beetles more vigorously than the dull manipulated beetles, resulting in haemolymph being exuded three times more often than for dull beetles (Table 2, Fig. 4b). Similar behaviour has previously been found in chickens; birds killed more aggregated aposematic prey than solitary prey^24, 25^, which suggests that birds, when recognizing potentially dangerous prey make a strategic decision to attack more forcefully. It is however worth noting that in our experiment 10 minutes was the maximum waiting time and the hesitation times were so long that the number of attacked beetles should be taken with caution, as it is unlikely an attack would occur in the wild after such a prolonged period of time.

**Table 2:** Number of birds in each trial that approached, attacked, killed or at least partly consumed the beetle during the predator assay.

Trial	Treatment							
	Dull Group				Glossy Group			
	Approach	Attack	Kill	Consume	Approach	Attack	Kill	Consume
1	1	11	4	0	1	4	10	0
2	10	3	1	1	9	5	2	0
3	13	3	0	0	11	2	3	0
4	14	1	1	0	12	3	1	0
Generalization Test	13	3	0	0	5	7	3	0

Data is divided between treatments and trials. Approach: indicates that the bird landed on the petri dish, excluding if the bird then attacked. Attacked: includes pecking, picking up and survivable damage (e.g. removing a leg), this excludes prey that was then killed. Killed: indicates that the bird has damaged the prey enough to release haemolymph, this does not exclude prey that was then consumed. Consumed: this indicates that a portion of the prey was eaten, it does not mean the whole prey was eaten.

After learning (Trials 1–4), we tested the birds’ generalization between glossy and dull beetles. The birds in the dull treatment generalized their learned avoidance of dull beetles to glossy ones and showed no difference in the attack latency between the last learning trial (Trial 4) and the generalization test (exp. coef. = 1.98, *z* = 0.78, *p* = 0.430, Fig. 4a). In the glossy group, the mean attack latency was reduced from 516 seconds (Trial 4) to 196 seconds (Generalization test): the birds did not transfer their learned avoidance of glossy beetles to dull beetles (exp. coef. = 20.68, *z* = 3.70, *p* = <0.001). In fact, attack probabilities for the first encounter of dull beetles and generalization test of glossy beetles were similar (exp. coef. = 0.97, *z* = −0.288, *p* = 0.819), strongly suggesting that there was no generalization from glossy to dull beetles.

## Discussion

We present experimental evidence of the role of specular reflectance and glossiness in warning signalling. We found that reducing the glossiness, hence the highlight, of *Oreina* beetle elytra influenced bird behaviour, leaving dull beetles more vulnerable to attack. Moreover, we found a strong asymmetrical generalization when bird’s experienced alternative prey. Birds readily learned to avoid both glossy and dull beetles at equal rates. However, birds that learned to avoid glossy prey readily attacked dull prey during the generalization test, whereas there was no difference in attack latency when birds first learned to avoid dull prey and were then presented with glossy prey. Our general conclusion is that glossiness augments aposematic learning, even if it is not critical for learning.

Our manipulation primarily reduced the intensity of specular reflectance of beetles whilst the tetrahedral colour space plot indicates that “colouration” of the elytra remained relatively constant (see also Fig. 1d, the shapes are similar). However, according to the avian visual model, the manipulation was distinguishable in both the chromatic and achromatic channels. The UV-absorbance of the matt varnish that we used to reduce specular reflectance caused only slight changes in shape of the reflectance spectra (Fig. 3b). The lights in our experimental aviaries provided almost no UV wavelengths (see Supplementary Fig. S2) therefore UV-absorbance of the warmish had little effect on beetle colouration in experimental conditions. Although it was not possible to replicate the viewing geometry exactly between the elytra measurements and the behavioural assays, the convex shape of the beetle guarantees the presence of specular reflectance regardless of the viewing geometry^2^. Our elytra measurements report the behaviour of light at a single point on the elytra, which is important for understanding our manipulation. However, the convex shape of *O. cacaliae* as a whole will always create viewing geometries optimal for maximum reflectance. While the positions of the chromatically rich highlight and the matt black regions of the elytra will change, each will be present regardless of viewing angle. This is markedly different to the ‘on-off’ signal created by the flat structurally coloured surfaces such as those of butterfly wings, and is to be expected of a continuous and convex interference colour with plates parallel to the curved surface.

*Oreina cacaliae* were highly unpalatable to blue tits and the pyrrolizidine alkaloids stored in the haemolymph of the beetle^14^ were sufficient to induce avoidance learning. Although the speed of avoidance learning did not depend on the manipulation, initial avoidance was stronger for glossy beetles. Ham *et al.* ^24^ identified similar behaviour in great tits (*Parus major*): despite some initial preference for grey and a hesitation to attack red prey, learning occurred at similar rates regardless of which colour signalled unpalatability in simultaneous presentations (yellow prey was also included). The palatability of prey was equal between treatments in our experiment, meaning that the learned association between palatability and appearance resulted in birds learning at a similar rate regardless of initial preference of birds. Whilst our experiment does not attempt to understand the cognitive processes behind this result it suggests that the cognition associated with a first encounter is separate from the cognition associated with learning.

While during the initial encounter signal intensity is important in the decision to attack; it may be the experience after the attack that determines the speed of learning. The initial avoidance of conspicuous, salient signals may balance the risk of detection associated with an increase in conspicuousness^26–28^ suggesting that naïve predators play a significant role in selecting for warning signals^29^. Given that the birds used in the experiment were wild caught, the latency to attack both beetle types (mean latency for dull: 43.38 s and glossy: 120.19 s) during the first trial may indicate an innate or previously learned avoidance. Indeed, although *Oreina* beetles do not co-occur with the Finnish blue tits we used as predators, there are glossy, chemically defended leaf beetles which may be within the range of the birds’ experience^25^, for example, *Linaeidea aenea*, which is smaller than *O. cacaliae* and utilizes very different chemical defences (isoxazolinone and nitropropanoic acid glucosides^30^). However, it is not known if birds can distinguish between these similar species when they are encountered in a different context. More importantly however, although the birds hesitated before attacking for a relatively long period of time during the first encounter, they further learned to avoid the beetles in the subsequent trials indicating that learning was by no means at asymptote. If the birds were familiar with this type of prey, it would be unlikely that there would have been such a clear learning pattern. For example, Exnerová *et al.* ^31^ using experienced wild caught blue tits, which co-occur with aposematic firebugs (*Pyrrhocoris apterus*), recorded an attack rate of only 22% during the experimental ‘first encounter’. In contrast to that, the blue tits we used attacked both the glossy and dull beetles 96% of the time.

The strong asymmetric generalization observed between treatments is often linked with peak-shift, where discriminative learning of a signal is generalized in one direction (positively or negatively), producing a peak shift of the stimulus response curve^32, 33^. Peak-shifts could cause signals to evolve in a certain direction, potentially triggering the suggested stepwise increase in conspicuousness (see ref. 34). Gamberale-Stille and Tullberg^33^ reported asymmetric generalization in experienced chicks with increasing strength of redness in seed bugs (Lygaeidae). Chicks avoided strongly red coloured bugs after having experienced distasteful bugs that were less red. However, chicks which first experienced bugs with the strong red colouration did not generalize their avoidance onto bugs with the weaker red colouration. Asymmetric generalization has been identified in other systems, for example great tits (*Parus major*) avoided red fire bugs (*Pyrrhocoris apterus*, wild type) after learning to avoid yellow and white bugs (mutant) of the same species but not the other way around^35^. Prey size has also produced similar asymmetric results where larger aposematic prey seems better protected than smaller prey^34^.

Green colours are commonly associated with crypsis^36^ and in many experiments used as a neutral colour with no initial avoidance biases by predators expected (e.g. refs 37, 38 and 39). However, many green insect species are thought to be aposematic (for example many Coleoptera) because of their utilisation of defensive chemicals, glossy appearance and bold behaviour^12, 40^. The most apparent difference between green cryptic and presumably aposematic species appears to be the specular reflectance (bright spot or highlight) caused by the glossy (smooth reflective) surface of the elytra. In some populations of *O. cacaliae* dull and glossy beetles can be found living side-by-side on the same host plant (personal observations SW, SD). At the same time *O. cacaliae* and other co-occurring *Oreina* species (e.g. *O. alpestris, O. gloriosa, O. speciosa, O. speciosissima*) are polymorphic in colour with green and blue representing the most common morphs. There is strong suggestion that the species locally form Müllerian mimicry rings^13^, as either green or blue colours dominate at specific sites with the Zastler site used here featuring almost exclusively green beetles (personal observations SD, SW). The signal value of these different colours will be investigated in future studies, but in the current context we first wanted to focus on the fact that for both colour morphs dull and glossy beetles can be found in all populations.

Our results suggest that predators can perceive the presence of specular highlights as a more salient warning signal when compared to a dull surface. Thus, glossy, highly reflective elytra may produce the most advantageous warning signal in alpine leaf beetles. This raises the question, how can both dull and glossy beetles co-exist in the same population and in such close proximity? A recent study by Fabricant and Herberstein^41^ discusses the consequences of a system with combined structural and pigment coloured aposematic prey and a predator with a monochromatic visual system (e.g. praying mantis and arachnids). They found that a classic aposematic colour (orange) acts as a camouflage against *Hierodula majuscule* (Mantidae), as its vision is reliant more on luminance contrast rather than chromatic contrast (see also ref. 9). Luminance contrast can be defined as the perceived difference between reflected light off an object and the background^9, 42^. Due to a reduction in luminance contrast it is possible that dull *O. cacaliae* appear less conspicuous to predators with monochromatic vision. It is therefore possible that variations in predator communities maintain the dull beetles found within certain *Oreina* populations. Indeed recent work by Valkonen *et al.* ^43^ and Nokelainen *et al.* ^44^ both show that variation in predator communities can play an important role maintaining the signal diversity in aposematic species as theorized and modelled by Endler and Mappes^45^.

Although we could not, with our experimental design, determine what specific cognitive processes caused the asymmetrical behaviour, our results suggest that glossy, highly reflective surfaces function as a stronger warning signal against bird predators. The convex shape of some beetles ensures that there is always the correct angle to create the glossy spot of maximum reflectance towards the viewer regardless of the angle of view. This means that the signal remains on, possibly reducing signalling costs. This may help explain the high frequency of glossy surfaces in aposematic insects and may also have aided in the evolution of warning signals by enhancing so called intermediate stages of increased conspicuousness. The next steps would be to determine if there is a stronger bias for glossy prey by creating a gradient in specular highlight intensity, similar to the colour gradient (yellow, orange, red) used by Ham *et al.* ^24^. Additionally, it is important to continue to isolate the individual effects of interference structural colours, most notably directionality but also chroma.

## Electronic supplementary material

Supplementary Information
