# Supplementary material for: Experimental evidence suggests that specular reflectance and glossy appearance help amplify warning signals
Source: Sci Rep. 2017 Mar 21;7:257. doi: 10.1038/s41598-017-00217-5 (PMC5427979; doi:10.1038/s41598-017-00217-5)
Supplement: Supplementary file 1 — Supplementary Information [file 41598_2017_217_MOESM1_ESM.pdf]

<u>Supplementary Information</u>

Experimental evidence suggests that specular reflectance and glossy appearance help amplify warning signals

Samuel J. Waldron, John A. Endler, Janne K. Valkonen, Atsushi Honma, Susanne Dobler and Johanna Mappes.

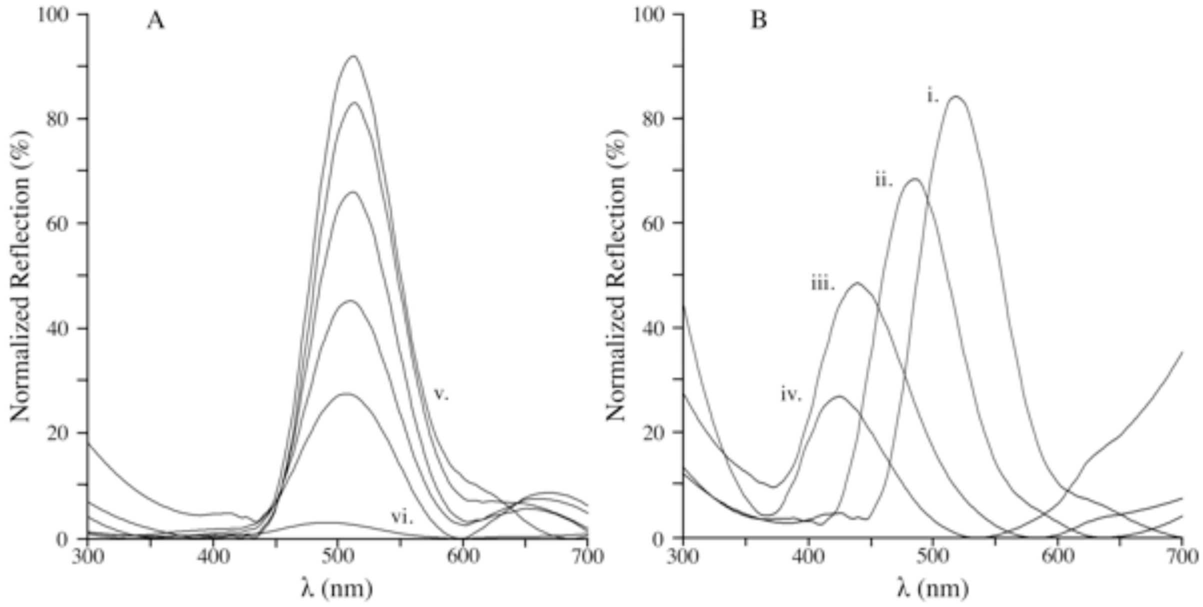

Figure S1. Structural colour of *O. cacaliae*. (A) Effect on reflectance when the ARM apparatus was fixed with a viewing angle of 40° (*i* and *r* = 20°) and the stage was tilted at 5° intervals from λ_{max} (-*t* = 3°). As the stage is tilted a large reduction in reflectance is seen to where the appearance is almost matt black (vi). Peaks represent spectra from (i) -*t* = 3°, (ii) -*t* = 8°, (iii) -*t* = 13°, (iv) -*t* = 18°, (v) -*t* = 23° and (vi) -*t* = 28°. When the samples were tilted away from the illumination (*t*) reflectance reduction occurred at a similar rate. (B) To measure a change in hue the ARM apparatus was rotated to produce a viewing angle of 30° where the angles of incidence (*i* and *r*) were equal (15°). The stage was then tilted and fixed at λ_{max}. The illumination and receiver arms were symmetrically rotated, *i* and *r* = (i) 15° (ii) 25° (iii) 50° and (iv) 75°. A substantial change in luminance occurs when the angle of the elytra segment is tilted by relatively small amounts. After tilting (-*t*) the segment by only 25° the reflectance peak becomes almost undetectable and the appearance of the surface becomes almost matt black (figure 1A-C (main text) and panel A, spectra vi). As is typical of interference colours, the peak shifts with viewing angle while the chroma and total luminance declines (figure S1B). Chroma shift is evident when an elytron segment is fixed at λ_{max} (-*t* = 3°) and the

elevation of both arms of the ARM apparatus are reduced in symmetrical increments. As the incident angles increased symmetrically, the λ_{max} decreases from 518 nm (spectra i) to 425 nm (spectra iv). To determine if a coloured surface is produced by iridescence, a change in luminance and chroma must occur when these strict and specific viewing geometries are applied.

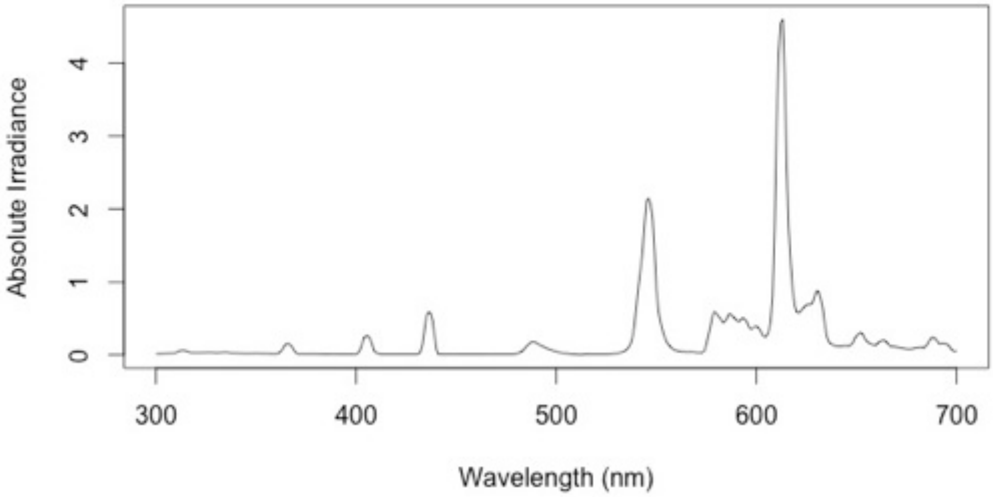

Figure S2. Absolute irradiance measurements (relative distribution of ambient light energy / wavelength) from the experimental arena used in the predation experiment. Note the virtual absence of UV in these experimental conditions. Light conditions in our experimental aviaries were measured using a cosine corrector CC-3 (Ocean Optics) positioned vertically where the beetles were placed during the experiment and attached to a 100µ optical fiber (Avantes). The set-up was calibrated for absolute irradiance measurement with DH-3 calibration light source (Ocean optics). Five absolute irradiance measurements were taken and averaged

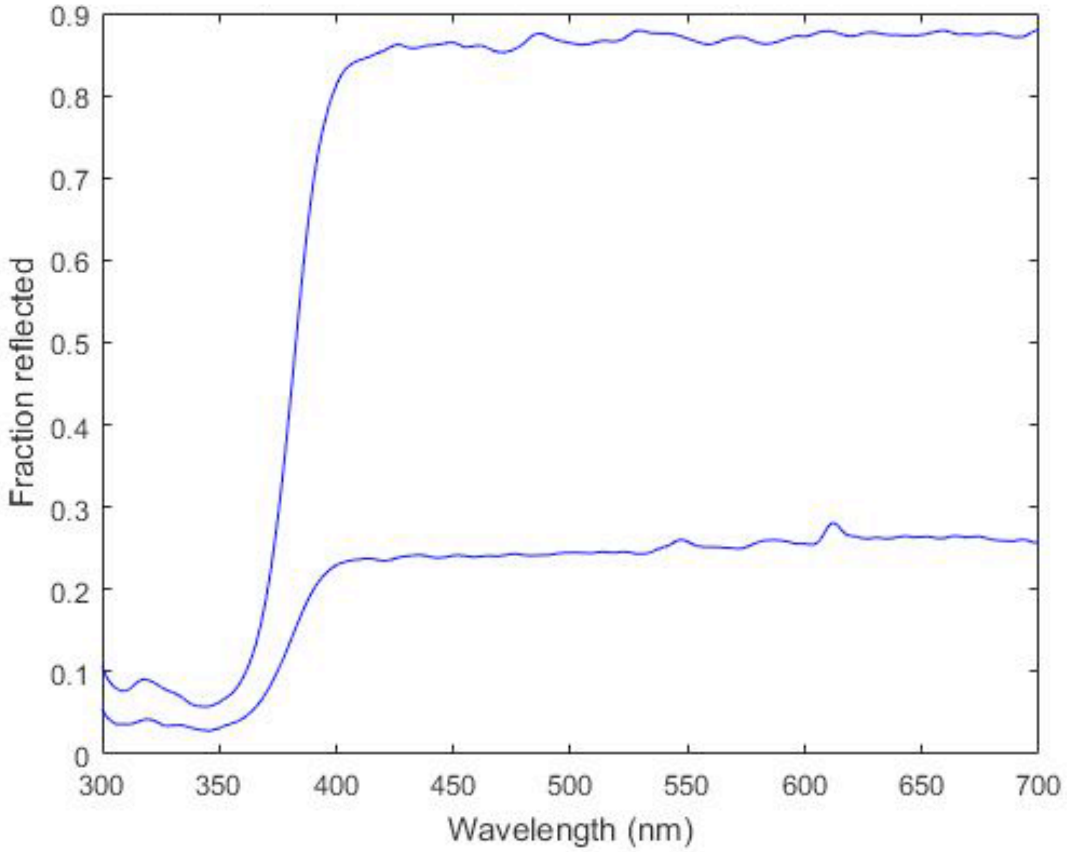

Figure S3. Reflectance spectrum of the varnish painted on a glass microscope slide and placed over a white standard (upper spectrum) and over a black standard (lower spectrum). Reflectance was measured by placing a varnished glass slide over a standard, and reflected light from the incident light source (at a 45 degree angle) was collected by the spectrometer. The lower spectrum represents the surface reflectance of the varnish and the upper spectrum represents light coming jointly from (a) the varnish surface and (b) light going through the varnish, reflected from the spectralon standard, and transmitted through the varnish. Both are spectrally flat except at the UV range, but there was almost no UV in the lights used in the experiment (see Fig. S2). Consequently the varnish on the beetle would have a negligible effect on the beetle's colour under the experimental light conditions. This also shows that possibly uneven finish caused by hand painting would have negligible effect on the colour.

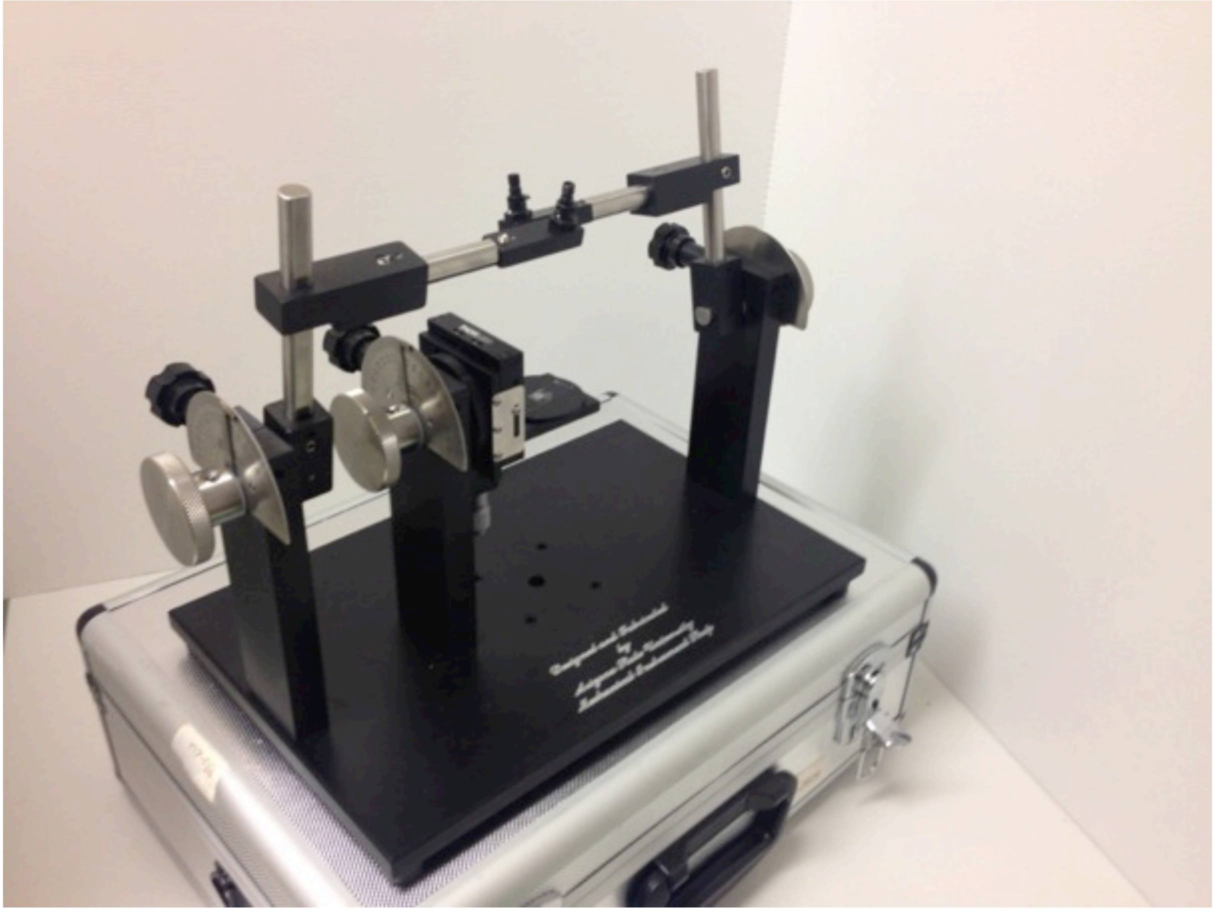

Figure S4. The ARM apparatus used for colour measurements is equipped with two rotating arms that could cover a range of 0° - 180° on the horizontal plane. The stage that held the sample could also rotate around the same axis perpendicular to the ARM. This allowed the sample to be tilted to establish maximum reflectance geometry and colour change. The stage could also be raised and lowered to allow for variations in the height of each sample to make sure the incident light and spectrometer aligned on the sample correctly. A change in the elytra reflectance was measured by fixing the ARM apparatus at a viewing angle of 40° ($i = r = 20°$) and tilting the stage (±t°) in 5° increments away from the maximum reflectance until the reflectance spectra was flat. To quantify the change in hue the ARM apparatus was rotated symmetrically so both incident angles ($i = r$) equalled 15°, the stage (t) was then tilted until the maximum wavelength (λ_{max}) was established for this viewing geometry ($-t = 3°$). Both arms were then rotated symmetrically and the reflectance was measured when i and r were equal to 15° (starting angle), 25°, 50° and 75°.

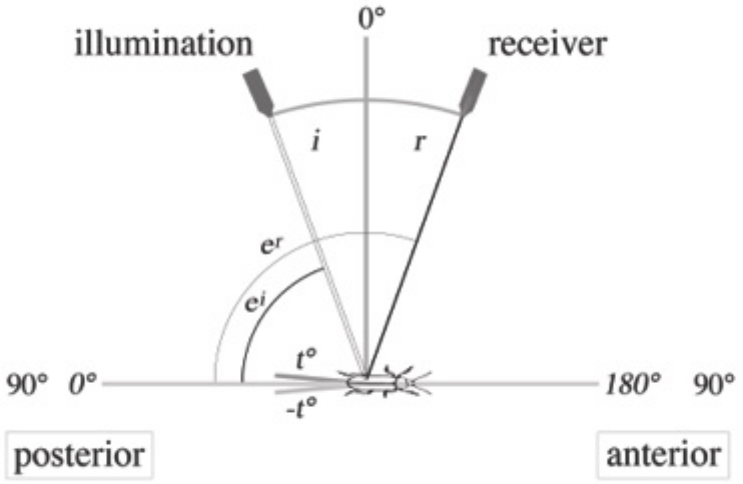

Figure S5. Schematic diagram of the viewing geometries used to investigate colour production in *O. cacaliae*. The elytron segment is positioned in reference to the position of the beetle on a posterior-anterior plane in relation to the azimuth of the illumination light source. To ensure illumination and receiver beams align on the sample, the stage can be raised or lowered (not indicated). The angles of incidence (incident angle (*i*) and reflectance angle (*r*)) are referred to when establishing the changes in luminance and chroma. Both angles are 0° when vertical and 90° when horizontal. The elevation (a.k.a. altitude) of the illumination (E_i) and receiver (E_r) refers to the inner angles on the posterior (0°) - anterior (180°) plane are referred to for all other measurements. Both the illumination and receiver arms can be positioned anywhere on the 180° posterior-anterior plane but cannot measure within ≈20° of one another due to the size of the apparatus, most notably between E_r = 55° and E_r = 75°. The viewing geometry used to ascertain λ_{max} was chosen to replicate a realistic scenario of an avian predator attacking a beetle at the time of collection. The sun was calculated to be at an elevation of E_i= 65° on the day and time when beetles are observed to be active and as λ_{max} is achieved when *i* and *r* are equal (1,2) the angle of an approaching predator was set at E_r = 115° (*i* and *r* = 25°). To establish λ_{max}, the beetle segment was tilted either towards the light source (-*t*°) or away from illumination (*t*°). The angle of the reflective surface of the elytra was found by tilting the stage in real time until λ_{max} was found, this is important as it may differ from the plane of the surface being measured (1,2). The λ_{max} was achieved by tilting the stage by 3° towards the light source (-*t* = 3°), meaning that the reflective surface of the elytra measured differs by 3° compared to the plane of the segment.

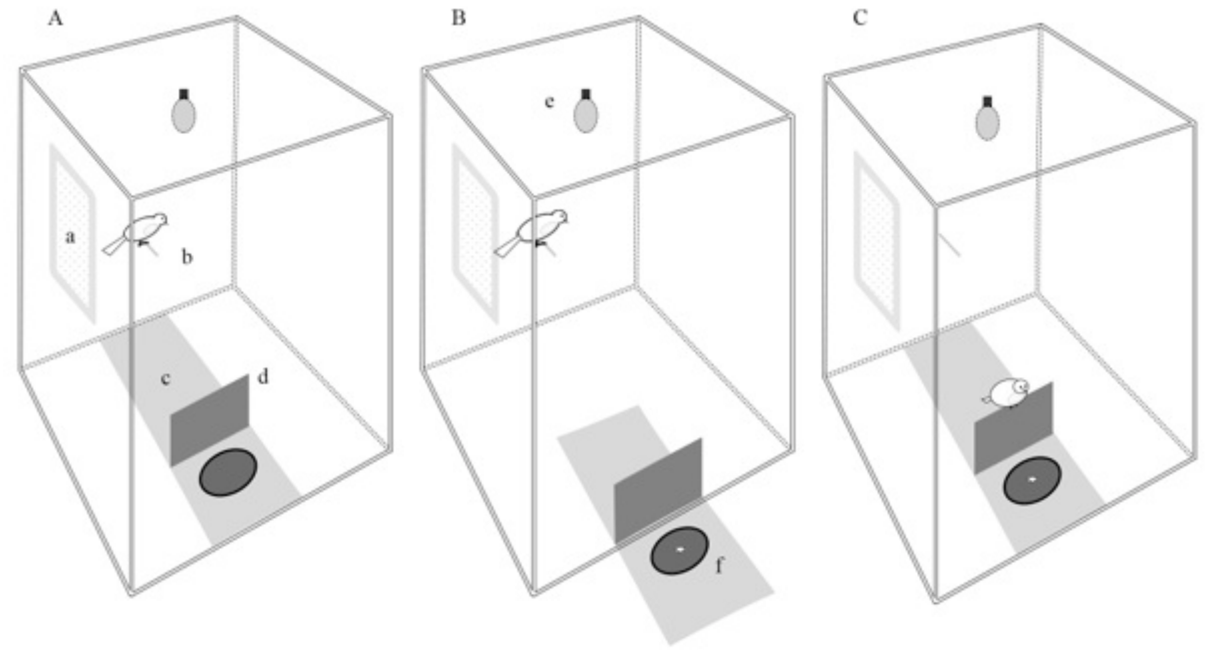

Figure S6. Schematic diagrams of the experimental aviaries, the observation hole (a), perch (b), sliding tray floor (c) and barrier (d) can be seen; the water bowl is not shown. The light bulb (e) is positioned in the center and is approximately 60 cm from the bottom of the arena. (A) The birds are trained to receive prey when they are positioned on the perch. (B) The sliding tray floor is retracted and the prey is placed on the petri dish (f) and positioned behind the barrier. The birds cannot see the prey from the perch. (C) As the sliding tray floor is returned to its original position the bird anticipates the prey from behind the barrier and lands on the barrier to investigate the prey. Each aviary had a perch, a water bowl (access *ad libitum*), a small mesh-covered observation hole and a moveable tray floor with a barrier attached.

Table S1. The maximum and minimum values for total reflectance (sum of reflectance over 300-700 nm range) and the angle (E_r) in which they were achieved.

		Non-manipulated		Manipulated	
		reflectance	angle (°)	reflectance	angle (°)
Elytra 1	Max.	7954	115	2526	115
	Min.	51	35	146	30
Elytra 2	Max.	11020	115	1985	120
	Min.	43	30	193	25
Elytra 3	Max.	15171	120	2390	115
	Min.	47	25	200	45
Mean	Max	11381 (2091)	115	2300 (162)	115
(s.e.)	Min.	43 (2)	30	180 (17)	35

Table S2. Comparing viewing angles (75°, 80°, 100° and 150°) identified in figure 2C of an approach and potential attack during the behavioral assay. Comparisons are made using the visual patch comparison model uses the same criteria described in the methods. Values are given in mean JNDs (Just Noticeable Differences) plus the standard error in brackets.

	Viewing angle comparisons					
Chromatic	75°-80°	75°-100°	75°-150°	80°-100°	80°-150°	100°-150°
Dull	1.8 (0.6)	3.4 (0.6)	8.1 (1.3)	2.5 (0.3)	6.7 (0.9)	5.2 (1)
Bright	1.6 (0.3)	3.5 (0.2)	7.7 (1.3)	2.2 (0.3)	7.1 (1.4)	7.3 (1.5)
Achromatic						
Dull	7.5 (1.5)	21.2 (0.7)	7.7 (1.9)	13.7 (0.8)	0.5 (0.1)	13.5 (1.2)
Bright	8.2 (2.9)	31.3 (2.5)	16.9 (2.2)	23.1 (0.5)	8.7 (1.3)	14.4 (0.9)

References

1. Osorio, D. & Ham, A.D. Spectral reflectance and directional properties of structural coloration in bird plumage. *J. Exp. Biol.* **205**, 2017-2027 (2002).

2. Meadows, M.G., Morehouse, N.I., Rutowski, R.L., Douglas, J.M. & McGraw, K.J. Quantifying iridescent coloration in animals: a method for improving repeatability. *Behav. Ecol. Sociobiol.* **65**, 1317-1327 (2011).
